# Deficient Insulin-mediated Upregulation of the Equilibrative Nucleoside Transporter 2 Contributes to Chronically Increased Adenosine in Diabetic Glomerulopathy

**DOI:** 10.1038/s41598-017-09783-0

**Published:** 2017-08-25

**Authors:** Sebastián Alarcón, Wallys Garrido, Génesis Vega, Claudio Cappelli, Raibel Suárez, Carlos Oyarzún, Claudia Quezada, Rody San Martín

**Affiliations:** Institute of Biochemistry and Microbiology, Science Faculty, Universidad Austral de Chile, Valdivia, Chile

**Keywords:** Permeation and transport, Diabetic nephropathy

## Abstract

Deficient insulin signaling is a key event mediating diabetic glomerulopathy. Additionally, diabetic kidney disease has been related to increased levels of adenosine. Therefore, we tested a link between insulin deficiency and dysregulated activity of the equilibrative nucleoside transporters (ENTs) responsible for controlling extracellular levels of adenosine. In *ex vivo* glomeruli, high D-glucose decreased nucleoside uptake mediated by ENT1 and ENT2 transporters, resulting in augmented extracellular levels of adenosine. This condition was reversed by exposure to insulin. Particularly, insulin through insulin receptor/PI3K pathway markedly upregulated ENT2 uptake activity to restores the extracellular basal level of adenosine. Using primary cultured rat podocytes as a cellular model, we found insulin was able to increase ENT2 maximal velocity of transport. Also, PI3K activity was necessary to maintain ENT2 protein levels in the long term. In glomeruli of streptozotocin-induced diabetic rats, insulin deficiency leads to decreased activity of ENT2 and chronically increased extracellular levels of adenosine. Treatment of diabetic rats with adenosine deaminase attenuated both the glomerular loss of nephrin and proteinuria. In conclusion, we evidenced ENT2 as a target of insulin signaling and sensitive to dysregulation in diabetes, leading to chronically increased extracellular adenosine levels and thereby setting conditions conducive to kidney injury.

## Introduction

Diabetes mellitus represents a major clinical problem in the occidental population with a predictable and dramatically high incidence^1^. One remarkable feature of diabetes is the metabolic imbalance due to deficient insulin production or inhibited signaling in target cells. Tissues and organs are damaged as a consequence of chronic diabetes, leading to complications such as diabetic nephropathy (DN)^2^. DN is the leading cause of end-stage renal disease worldwide that remains incurable and is source of morbidity and mortality^3^. Major efforts to understand the pathogenesis and derive therapeutic alternatives are ongoing as current approaches, using blockers of the renin angiotensin system and strict metabolic control, seem insufficient^4^.

Glomerular alterations in DN concur with podocyte dysfunction and altered glomerular filtration rate and permeability^5^. Profibrotic activation of cells and accumulation of extracellular matrix are also remarkable features that evolve to glomerulosclerosis^6^. It has been recognized to glomerular podocytes, mesangial and endothelial cells as insulin responsive entities^7–9^. Furthermore, there is data suggesting that insulin resistance or alterations affecting insulin signaling in these cells trigger glomerular alterations that resemble diabetic glomerulopathy^10–14^. Conversely, pancreatic islet transplantation has shown to, in the long term, reverse kidney injuries in type 1 diabetics or related experimental models^15^. Thus, the impact of insulin in controlling glomerular function is of great interest.

Recently, it was shown that disparate adenosine levels in plasma of DN patients is concurrent with progression of the disease^16, 17^. Further, experimental models of chronic kidney disease demonstrated increased extracellular levels of adenosine^18, 19^. Adenosine is an endogenous nucleoside whose extracellular levels can be transiently modified in the body compartments to effects physiological responses by signaling through adenosine receptors A_1_, A_2A_, A_2B_ and A_3_ subtypes^20^. In addition, stressor and pathological conditions such as epilepsy, ischaemia, pain, inflammation, and cancer has been related to persistent elevated levels of adenosine thus affecting its signaling properties and cells functioning^20, 21^. Further, the adenosine receptors have been implicated in modulate glucose homeostasis and lipid metabolism, insulin secretion and resistance in diabetes^22^. Extracellular levels of adenosine may be controlled by the activity of ecto enzymes that hydrolyze precursor nucleotides, and also by the uptake activity mediated by nucleoside transporters^18^. Nucleoside and nucleobase transporters are classified into two structurally unrelated protein families: the SLC28 sodium-dependent concentrative nucleoside transporters (CNTs) family and the SLC29 sodium-independent equilibrative nucleoside transporters (ENTs) family^23^. In rat glomeruli, the crucial role of the sodium-independent equilibrative nucleoside transporters-1 and -2 (ENT1 and ENT2) contributing to regulation of extracellular adenosine levels was elucidated^24^. Interestingly, higher levels of adenosine were found in urine and plasma of insulin deficient diabetic rats, supposing a role for hyperglycemia and insulin in adenosine handling by cells^25–27^. In fact, exposure of cells to high D-glucose was associated with decreased ENT1 uptake activity^25^.

Relevant roles for adenosine on renal physiology have been determined. Such is the case of the A_1_ receptor subtype on afferent arterioles to mediate tubule-glomerular feedback^28^, or in proximal tubules to regulate sodium reabsorption^29^. It was also shown the relevance of ENT1 and adenosine receptors on the process of reestablishing renal perfusion following ischemia in acute kidney injury^30^. However, the critical role of chronically increased adenosine and progression of kidney injury has only recently been recognized^18, 19^. Thus, the study of processes leading to dysregulation of extracellular nucleoside levels is a topic of great interest.

The aim of this research was to determine the role of glucose and insulin on control of ENT1 and ENT2 activity in the glomerulus. In addition, we demonstrated that dysregulation mediated by insulin deficiency creates conditions conducive to diabetic glomerulopathy.

## Results

### Extracellular level of adenosine is controlled by insulin through ENT2 upregulation

**Figure 1:**
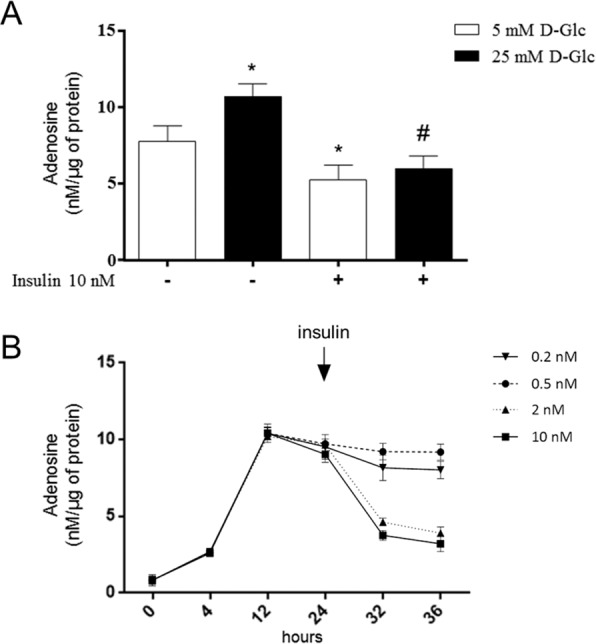
Increased adenosine induced by high D-glucose is restored by insulin in rat glomeruli. (**A**) Purified glomeruli from healthy male rats were exposed *ex vivo* to 5mM or 25mM D-glucose (D-glc) and insulin 10 nM for 24 h. Extracellular adenosine was quantified in aliquots of the medium by using derivatization with chloroacetaldehyde and HPLC. Values are means ± S.D. from individuals determinations normalized to 1 µg of total glomerular proteins. *P < 0.01 vs 5mM; ^#^*P* < 0.01 vs 25mM D-Glc; n = 6. (**B**) Time course of extracellular adenosine levels in glomeruli of rats exposed *ex vivo* to 25mM D-glucose and supplementation with insulin at 0.2, 0.5, 2 and 10nM at 24 h. Adenosine was quantified as described above n = 6.

The expression of nucleoside transporters from ENT and CNT families has been previously shown in the rat kidney glomerulus^24, 31^. Transport activity assays revealed the activity of sodium-independent equilibrative nucleoside transporters -1 (ENT1) and -2 (ENT2) account for more than 75% of total nucleoside uptake in rat glomeruli^24^. Whereas a decreased activity of ENT1 was a remarkable feature in glomeruli exposed to high D-glucose conditions *ex vivo* and in glomeruli isolated from experimental diabetic rats^24^, the contribution of ENT2 to adenosine handling in this compartment is much less known. In *ex vivo* assays we detected that extracellular adenosine was increased by exposure of isolated glomeruli to high D-glucose conditions, while insulin decreased adenosine level (Fig. 1A). The effect generated by insulin was dose-dependent and produced a significant decrease in adenosine level at 2 and 10 nM (Fig. 1B), which are doses equivalent to the upper physiological insulin range in rats^32^. Thus, clearance of glomerular adenosine over time supposes a role for insulin to regulate adenosine uptake by cells through ENT transporters consequently counteracting decreased uptake caused by high D-glucose.

**Figure 2:**
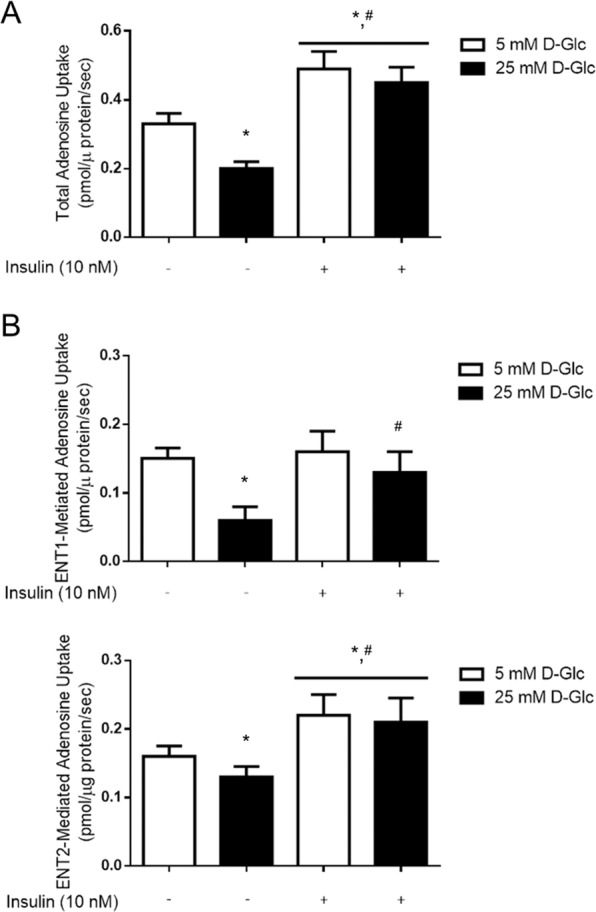
D-glucose and insulin affect adenosine uptake activity mediated by ENT1 and ENT2 in rat glomeruli. (**A**) Total adenosine uptake (10 μM adenosine, 60 seconds, 22 °C) mediated by sodium-independent transporter systems was measured in purified glomeruli from healthy male rats exposed to 5mM or 25 mM D-glucose (D-Glc) for 24 h and supplemented with insulin the last 30 minutes. (**B**) The effect of D-glucose and insulin treatments on particular uptake activity mediated by ENT1 and ENT2 are shown. ENT1-mediated uptake is the fraction of total adenosine transport in sodium-free buffer sensitive to NBTI and ENT2-mediated uptake is the fraction of total transport inhibited by hypoxanthine. ENT1-mediated uptake was the fraction of total adenosine transport in sodium-free buffer sensitive to NBTI and ENT2-mediated uptake was the fraction of total transport inhibited by hypoxanthine. The graphs represent the means ± S.D. *P < 0.05 versus 5 mM D-Glc; ^#^*P* < 0.05 vs 25mM D-Glc n = 9.

We characterize the effect of insulin on transport activity mediated by ENT1 and ENT2 in *ex vivo* glomeruli. Total sodium-independent adenosine uptake mediated by equilibrative nucleoside transporters, assayed in choline buffer (see methods), was decreased in glomeruli exposed to 25mM D-glucose conditions, compared to 5mM D-glucose (Fig. 2A). Osmotic control with D-mannitol has no effect on transport activity (Supplementary Fig. S1). Insulin (10nM) increased total adenosine uptake in *ex vivo* glomeruli irrespective D-glucose concentration (Fig. 2A). Particular uptake rates mediated by ENT1 or ENT2 subtypes could be individualized through selective inhibition with 1 µM S-(4-nitrobenzyl)-6-thio-inosine (NBTI) or 2 mM hypoxanthine, respectively. Thus, ENT1-mediated uptake is the fraction of total adenosine transport in sodium-free buffer sensitive to NBTI and ENT2-mediated uptake is the fraction of total transport inhibited by hypoxanthine. We found both transporters contributed similarly to overall adenosine uptake in rat glomeruli, and their activities were both inhibited by exposure to high D-glucose with ENT1 decreased more than 50% (Fig. 2B). However, insulin has differential effects on these transporters. While restoring partially the decreased activity of ENT1 due to high D-glucose, there is a correlation between the presence of the insulin and increased ENT2-dependent uptake regardless of D-glucose concentration (Fig. 2B). We thereby conclude that insulin-triggered uptake activity mediated by ENT2 is a major contribution to restoration of basal levels in extracellular adenosine.

### Insulin receptor signaling by PI3K pathway increases ENT2 activity

**Figure 3:**
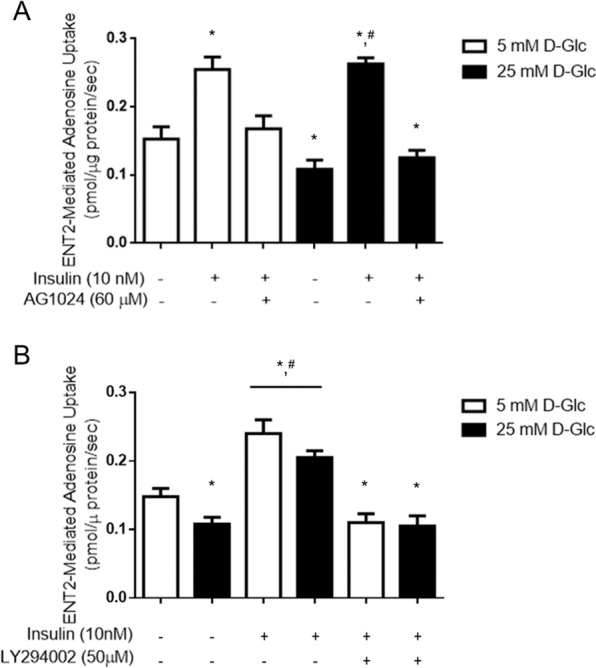
ENT2-dependent uptake is increased by insulin receptor signaling through PI3K in rat glomeruli. ENT2-mediate uptake (10 μM adenosine, 60 seconds to 22 °C) was measured in purified glomeruli from healthy male rats exposed to 5mM or 25 mM D-glucose (D-Glc) for 24 h and supplemented with insulin the last 30 minutes. Signaling through insulin receptor and intracellular pathways were characterized by using AG1024 inhibitor of the tyrosine kinase activity of the receptor and LY294002 PI3-kinase inhibitor. Graphs represent the means ± S.D. *P < 0.05 versus 5 mM D-Glc; ^#^*P* < 0.05 vs 25mM D-Glc n = 9.

We demonstrated that using the inhibitor of insulin receptor (IR) kinase activity AG1024, the increased activity of ENT2 mediated by insulin was impeded, thus confirming the selective role of this receptor on nucleoside uptake (Fig. 3A). Further, we explored intracellular pathways downstream IR related with ENT2 regulation. Using an inhibitor of PI3K activity, we demonstrate this axis was necessary to mediate ENT2 upregulation (Fig. 3B). We conclude ENT2 is a target of insulin signaling in rat glomeruli.

### Insulin affects kinetic parameters of ENT2

**Figure 4:**
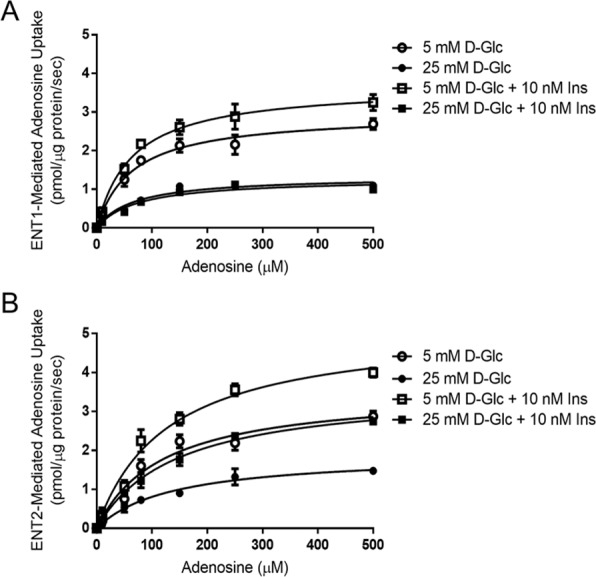
Kinetics of adenosine uptake mediated by ENTs are affected by D-glucose and insulin. Primary cultured podocytes were exposed to 5mM or 25mM D-glucose for 24 h and supplemented with 10nM insulin the last 30 minutes. Adenosine transport activity (0–500 µM adenosine, 60 seconds to 22 °C) mediated by ENT1 (**A**) and ENT2 (**B**) were measured. Data were adjusted to Michaelis-Menten equations without a lineal component. Graphs represent means ± S.E. n = 9.

Primary cultured rat podocytes were used to evaluate effects of insulin on ENT2 transport properties. These analyses showed decreased maximal velocity of adenosine transport (*V*_max_), mediated by both ENT1 and ENT2, when podocytes were exposed to high D-glucose condition (Table 1 and Fig. 4). Presence of insulin was correlated to an increase of *V*_max_ for ENT2, and somewhat for ENT1, when transport was assayed in 5mM D-glucose, however only *V*_max_ of ENT2-mediated transport of adenosine was increased by insulin under high D-glucose conditions (Table 1 and Fig. 4). Kinetic parameters of transporters ENT1 and ENT2 shown in Table 1 indicate that transport capacity of both transporter systems (*V*_max_/K_m_) were decreased by high D-glucose and only transport capacity through ENT2 was recovered by insulin.

**Table 1:** Kinetic parameters of adenosine transport mediated by ENT1 and ENT2 in rat podocytes.

	*V*_*max*_ (pmol/µg protein/sec)	K_m_ (µM)	*V*_*max*_/K_m_
**ENT1**			
5 mM D-Glucose	2.955 ± 0.1870	65.05 ± 13.73	0.045
25 mM D-Glucose	1.333 ± 0.0829^a^	66.74 ± 13.71	0.020^a^
5mM D-Glucose + insulin	3.678 ± 0.2044^b^	64.31 ± 11.98	0.057
25mM D-Glucose + insulin	1.279 ± 0. 0930^a^	73.77 ± 17.03	0.017^a^
**ENT2**			
5mM D-Glucose	3.544 ± 0.3037	119.6 ± 27.10	0.030
25mM D-Glucose	1.913 ± 0.1828^a^	137.1 ± 32.98	0.014^a^
5mM D-Glucose + insulin	5.170 ± 0.3442^b^	128.1 ± 22.00	0.040
25mM D-Glucose + insulin	3.576 ± 0.2321^c^	144.1 ± 23.11	0.025^c^

Primary cultured podocytes were exposed to 5mM or 25mM D-glucose for 24 h and supplemented with 10nM insulin the last 30 minutes.

Adenosine transport was assayed at 0–500 µM adenosine, 60 seconds to 22 °C. Graphics depicting adenosine uptake mediated ENT1 and ENT2 are in Fig. 1 of supplementary material.

Data represent means ± S.D. ^a^*P* < 0.05 versus 5 mM D-Glucose. ^b^*P* < 0.05 versus 5mM D-Glucose. ^c^*P* < 0.05 versus 25 mM D-Glucose. n = 9 independent experiments each one in duplicates.

### Insulin deficiency decreases ENT2 in the diabetic kidney

**Figure 5:**
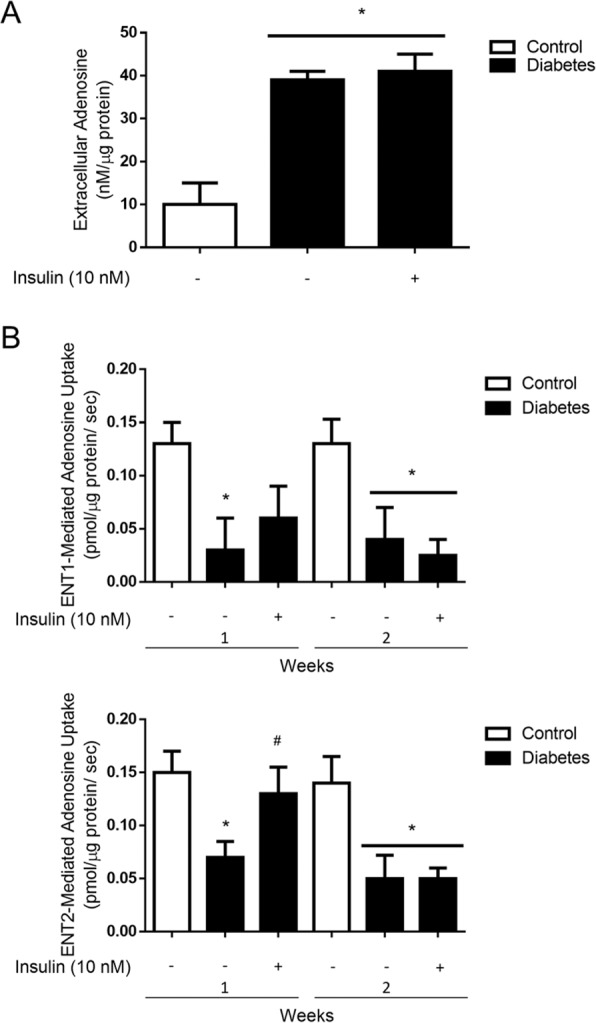
Upregulation of ENT2 by insulin is impaired in glomeruli from diabetic rats. (**A**) Freshly purified glomeruli from healthy control and diabetic rats were incubated in Tyrode’s buffer for 1 min and extracellular adenosine was quantified. The graph represents means ± S.D. from individuals determinations normalized to 1 µg of total glomerular proteins. *P < 0.01 vs control; n = 5 animals in each group. (**B**) Particular adenosine uptake mediated by ENT1 and ENT2 (10 μM adenosine, 60 seconds to 22 °C) was measured in purified glomeruli from healthy control male rats and diabetic rats. The graphs represent the means ± S.D. *P < 0.05 versus control; ^#^*P* < 0.05 versus diabetes; n = 6 animals in each group.

Long term effects of insulin deficiency on adenosine levels and ENTs activity were evaluated in streptozotocin (STZ) treated rats, a diabetic model resulting from depletion of pancreatic beta cells. In this model, extracellular levels of adenosine in glomeruli isolated from STZ-induced diabetic rats were significantly increased compared to adenosine levels in control rats^24^. This alteration was caused by a metabolic imbalance due to insulin deficiency; since insulin replacement therapy in STZ-induced diabetic rats (sufficient to attenuate hyperglycemia) produced glomeruli with extracellular adenosine levels similar to those found in non-diabetic animals (Supplementary Fig. S2). To evaluate if adenosine transport capability were affected, following 7 and 15 days from diabetes induction glomeruli were isolated and exposed *ex vivo* to insulin. Levels of extracellular adenosine were increased in diabetic glomeruli and remarkably, exposition to insulin *ex vivo* did not restore basal levels (Fig. 5A). These facts evidenced that diabetes altered adenosine uptake activity in response to insulin in glomeruli. Indeed, uptake activity mediated by ENT1 was severely affected as early as one week from diabetes induction while, insulin-mediated ENT2 upregulation of transport activity was hampered at this stage and completely lost at day 15 (Fig. 5B). These changes were sustained over time and urinary adenosine levels were significantly increased in urines from diabetic rats following 30 days post diabetes induction (22 ± 8 µmoles • g^−1^ creatinine in normal rats versus 66 ± 18 µmoles • g^−1^ creatinine in diabetic rats).

**Figure 6:**
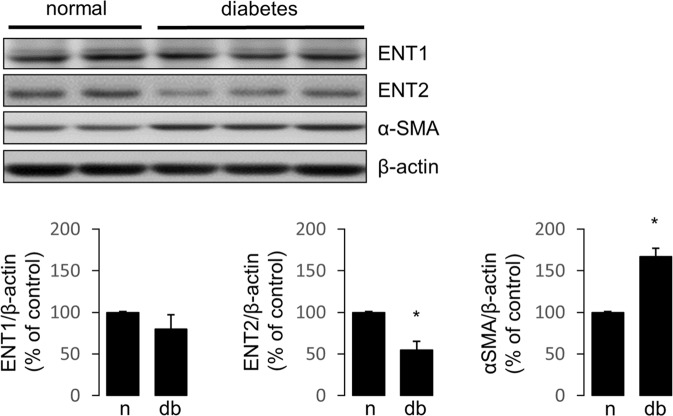
ENT2 is downregulated by diabetes. The protein levels of ENT1 and ENT2 were evaluated in the renal glomerulus of normal and diabetic rats following 30 days of diabetes induction. Representative western blots analysis in protein extracts from purified glomeruli from rats. Induction of α-SMA was included as marker of glomerular dysfunction by diabetes and β-actin as loading control. For western blots the membrane from each gel was cut in the pieces comprising proteins in the range of 45–70 kDa and 10–45 kDa. ENT1 and ENT2 were consecutively detected in the upper piece using an stripping procedure. Similarly, α-SMA and β-actin were consecutively detected in the piece containing lower molecular weight proteins. Graphs show means ± S.D. from quantitative results from western blot analyses. Values of normal samples were normalized to 100. *P < 0.05 vs normal; n = 5 animals in each group.

**Figure 7:**
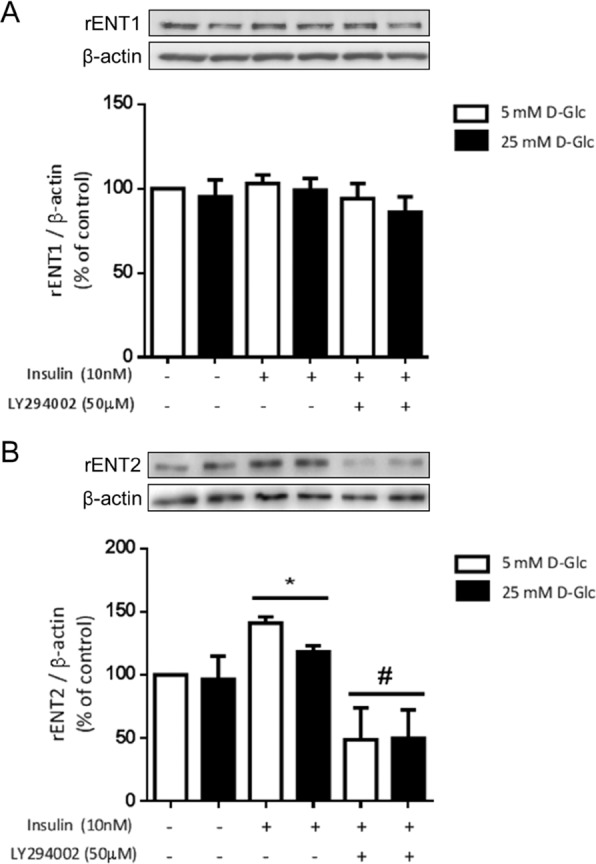
Protein levels of ENT2 are downregulated by inhibition of PI3K signaling pathway in podocytes. Primary cultured podocytes were exposed to insulin and LY294002 PI3-kinase inhibitor for 48 h. Representative western blot analyses of ENT1 (**A**) and ENT2 (**B**) are shown. For western blots the membrane of each gel was cut in pieces comprising proteins in the range of 45–70 kDa and 10–45 kDa. ENT1 or ENT2 were detected in the upper piece and β-actin in the respective piece containing lower molecular weight proteins. Graphs represent the means ± S.D. *P < 0.05 versus 5 mM D-Glc; ^#^*P* < 0.05 vs insulin; n = 5.

Western blot analyses showed ENT2 expression level reduced in glomeruli from diabetic rats (Fig. 6). In contrast, the expression levels of α-SMA, a marker of profibrotic activation of cells, was increased and ENT1 was not significantly affected (Fig. 6A). Therefore, the role of insulin signaling on ENT1 and ENT2 expression was evaluated using primary cultured podocytes. Following 48 h of treatments, ENT1 expression levels were not affected by exposition to high D-Glucose, insulin or PI3K inhibitor (Fig. 7A). However, ENT2 expression was increased following exposition to insulin for 48 h (Fig. 7B). Notably, inhibition of PI3K signaling pathway significantly decreased ENT2 expression level (Fig. 7B). Thus, we conclude that insulin/PI3K signaling seem necessary to maintain ENT2 expression levels.

### Deficient adenosine uptake and increased adenosine levels are related to renal injury in diabetes

**Figure 8:**
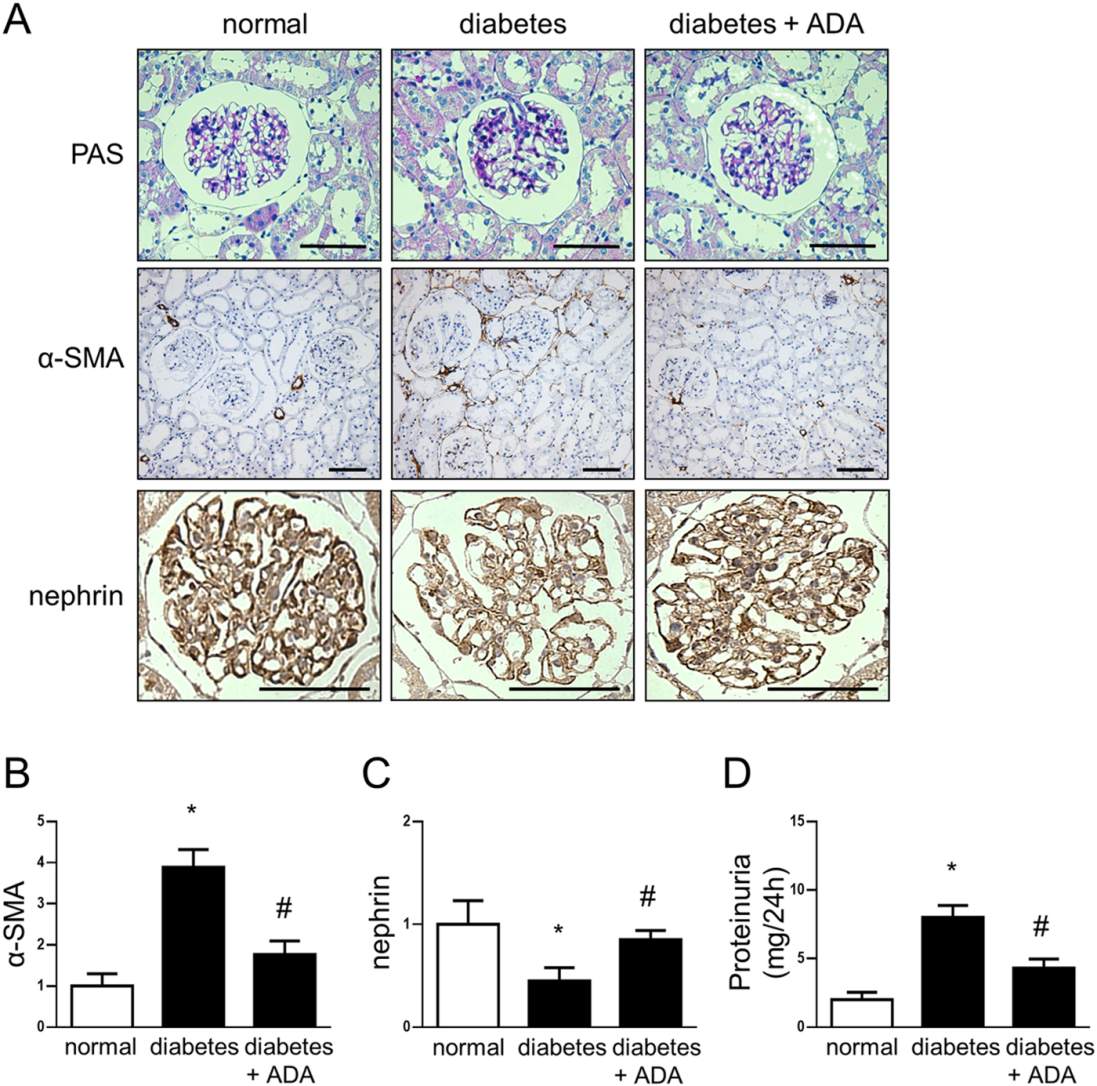
Increased adenosine level is correlated with early kidney alterations in diabetes. Following 1 month from diabetes induction rats were treated with PEGylated adenosine deaminase (ADA) for four weeks to decrease adenosine levels. (**A**) Representative images of the Periodic Acid-Schiff (PAS) staining and the immunohistochemical detection of α-SMA and nephrin in the kidney from normal rats and diabetic rats untreated or treated with ADA are shown. Bar indicates 50µm. Original magnifications 400X. (**B**,**C**) Graphs show quantitative analysis of α-SMA and nephrin staining in rat renal samples. Each animal group contained 5 rats. A number of 20 images captured from renal slides of each rat were used to quantitative analysis using the UN-SCANIT 2.0 software. *P < 0.01 vs normal; ^#^*P* < 0.01 versus diabetes; n = 5. (**D**) The graph represents the means of urinary protein levels in rat groups at end point follow up. *P < 0.01 vs normal; ^#^*P* < 0.01 versus diabetes; n = 5.

Our observations about deficient adenosine uptake in diabetic glomeruli may be correlated with increased extracellular levels of adenosine. Thus, dysregulated nucleoside uptake may provide the conditions that lead to diabetic kidney disease. To demonstrate a deleterious effect of increased adenosine on the kidney we evaluated the administration of adenosine deaminase (ADA) enzyme, which converts adenosine to inosine thus finishing its biological effects, on renal injuries in diabetic rats. Treatment of diabetic rats for four weeks with ADA decreased the chronically augmented adenosine, resulting in urinary adenosine levels comparable to those found in normal rats (25 ± 8 µmoles • g^−1^ creatinine). Furthermore, ADA treatment attenuated the loss of glomerular nephrin and reduced proteinuria in diabetic rats, which correlated with a protective effect against diabetic glomerulopathy. Also, the induction of glomerular and tubulointerstitial α-SMA, a profibrotic marker, was decreased in diabetic rats treated with ADA (Fig. 8).

## Discussion

Extracellular adenosine homeostasis is dependent on a finely regulated uptake process by glomerular cells through nucleoside transporters. Meanwhile, disparate levels of the nucleoside were evidenced during experimental diabetes due to decreased transport activity mediated by ENTs in the glomerulus, without affecting CNTs subfamily transport capacity^24^. The present study demonstrates that ENT2 is a target of insulin actions at the glomerulus, and dysregulated control of this transporter is linked to increased levels of adenosine. ENT2 is a low affinity nucleoside transporter with an apparent K_m_ higher than ENT1 and CNTs^33^, accordingly upregulation of its activity by insulin could represent a relevant mechanism to clearance extracellular adenosine under a condition with a high nucleoside level as occur in glomeruli exposed to high glucose concentration. Consequently, insulin counteracting high D-glucose effects contributes to the restoration of basal adenosine levels at this compartment by switching nucleoside uptake activity predominantly mediated by ENT2. Importantly, balance of this regulatory mechanism may be sensitive to alterations that characterize uncontrolled diabetes such us insulin deficiency, insulin resistance and persistent hyperglycemia.

It is well known that insulin resistance and poor glycemic control is present in type 1 and type 2 diabetic patients, being a risk factor for the development of diabetic kidney disease^10–12^. At the glomerular cells, some effects due to impaired insulin signaling has been described with most of these studies achieved in podocyte^34^. A study in rats conducted by Mima and col.^35^ demonstrated insulin activated the IRS1/PI3K/Akt pathway in both the glomeruli and tubules; however analyzing both a type 1 diabetes model generated by STZ treatment and a type 2 diabetes model by using fatty Zucker strain, it was observed the loss of insulin signaling exclusively in glomeruli, suggesting that these cells are more susceptible to developing insulin resistance. Similarly, significantly diminished insulin responses were described, with a loss of insulin receptor and PI3K/Akt pathway signaling occurring in podocytes isolated from db/db mice^36^. Furthermore, by inducing insulin resistance it was evidenced a more severe glomerular phenotype in the db/db model of diabetes^37^. On the other hand, we recently demonstrated downregulation of insulin receptor protein in kidney cortex from STZ induced diabetic rats and diabetic patients^38^. Thus, insulin deficiency such as in STZ diabetic rats or deficient IRS1/PI3K/Akt signaling as occurs in insulin resistance, may affect adenosine handling by downregulating nucleosides uptake leading to chronically increased levels of adenosine. On the other hand, our results propose that tightly adjusted metabolic control, when possible, may contribute to the maintenance of homeostatic levels of glomerular adenosine; since extracellular adenosine levels were reestablished, at least partially, in diabetic rats that received insulin replacement therapy.

In addition to upregulation of ENT2, we also found that decreased activity of ENT1 that occurs in high glucose conditions could be partially recovered by insulin in normal glomeruli *ex vivo*. Some reports recognize insulin actions on counteracting decreased nucleoside uptake activity imposed by elevated D-glucose, through ENT1 activity rescue and increasing ENT2-mediated transport in human endothelial cells and rat lymphocytes^39–42^. One of these studies also proposed a role for insulin to restore increased extracellular levels of adenosine into umbilical vein, thus finishing pathological purinergic effects by signaling through adenosine receptors^42^. In the context of diabetic kidney disease, it has been shown chronically increased levels of adenosine at the kidney, plasma and urine of STZ-treated rats^24–27^. In humans, increase of plasma adenosine levels with progression of the disease was described in patients affected by diabetic nephropathy in recent years^16, 17^. In turn, purinergic signaling may contribute to progression of chronic renal disease. Remarkably, the adenosine deaminase knockout (*ada*^*−/−*^) mice model, exhibiting augmented levels of the nucleoside because of impeded metabolism, shows renal dysfunction and sclerosis that resemble diabetic nephropathy^43^. Consequently, protective effects of antagonists of low affinity adenosine receptors occurring in renal cells types have been evaluated. Indeed, Dai and col^43^. demonstrated that development of renal fibrosis generated in *ada−/−* animals can be avoided using an A_2B_AR antagonist. Other works demonstrated the induction of the A_2B_AR receptor subtype related to podocyte cell dysfunction, increased TGF-β release and diabetic glomerulosclerosis^24, 26^ and signaling through A_3_AR involved with mesenchymal-like transition of tubular epithelial cells^25^. Therefore, using adenosine deaminase to attenuate proteinuria and reduce α-SMA distribution in diabetic rats is additional evidence that further supports the role of chronically altered nucleoside handling in producing renal injury. Interestingly, using different experimental approaches, it has also suggested that adenosine increase and signaling is a common pathogenic pathway in chronic kidney injury^43^. Such is the case of renal fibrosis in mice generated by angiotensin II infusion and unilateral ureteral obstruction (UUO)^43, 44^. Whereas failed adenosine uptake due to deficiency of insulin was recognized in our diabetic model, the mechanism leading to chronically high adenosine levels in these other models of chronic kidney disease remain to be determined.

Pawelczyk and col.^45^ first described downregulation of ENTs coding mRNAs in the diabetic kidney with major effects on ENT2 mRNA. Interestingly, they demonstrated that administration of insulin in STZ-diabetic rats resulted in a drop in adenosine concentration in examined tissues and concomitantly returned ENT2 mRNA levels to values observed in nondiabetic animals. Additionally, it was evidenced that increased expression and activity of ENT2 as a consequence of insulin in rat cardiac fibroblast^46^, lymphocytes^40^ and human hPMEC cells^47^. The mechanisms implicated in regulation of ENTs expression or activity have been scarcely investigated at the cellular level. We found changes in transporters *V*_*max*_ thus contributing to homeostatic balance of extracellular adenosine. Changes in *V*_max_ could be caused by an alteration in transport efficiency, number of transporters at the plasma membrane or both. Some studies have evidenced that dual distribution of ENT1 and ENT2 into intracellular compartment and plasma membrane^48, 49^. In addition, Bone and col.^50^ demonstrated ENT1 as having a casein kinase 2 (CK2) phosphorylation site (Serine 254). The inhibition of CK2 decreased *V*_*max*_ and reduced ENT1 transporter at plasma membrane^50, 51^. Meanwhile, regulation of ENT1 by CK2 activity remains debatable^52^, much less information exists regarding ENT2 regulation. Thus, further research will be necessary to links IR/PI3K signaling with ENT2 subcellular distribution or transport efficiency in the kidney.

In conclusion, we found that insulin targeting ENT2 activity is crucial to homeostatic control of adenosine levels in the glomerulus. Further, our contribution is recognizing the activity of nucleoside transporters being sensitive to the metabolic and endocrine imbalance in diabetes resulting in setting the conditions for a pathogenic adenosine signaling.

## Methods

### Animals and sample collection

Diabetes was induced in male rats (Sprague-Dawley) weighing 250 g by single intravenous administration of streptozotocin (STZ) at 65 mg/kg dissolved in citrate buffer, pH 4.5^24^. Control rats were injected with an equivalent volume of vehicle. The diabetic groups included animals presenting blood glucose levels ≥ 25 mmol/L. Urine and renal tissue samples were collected from rats after 1 and 2 weeks post diabetes induction. Kidneys were used for glomeruli isolation or processed to histological analysis. All animal procedures were according to the Guide for the care and use of laboratory animals, Eighth edition (2011) (http://grants.nih.gov/grants/olaw/guide-for-the-care-and-use-of-laboratory-animals.pdf) and approved by the Institutional Committee on the Use of Live Animals in Research at the University Austral de Chile (Ref. 2012/59).

### Glomeruli isolation

Renal cortex of male rats (Sprague–Dawley) weighing 200–250 g was sieved through 212 μm, 150 μm, 106 μm and 75 μm meshes. The material collected with the narrowest sieve corresponded to glomeruli with purity higher than 90%^53^.

### Primary Culture of Podocytes

Isolated glomeruli were initially cultured on fibronectin coated culture dishes in DMEM:F12 medium supplemented with 10% v/v fetal calf serum, 100 units/ml penicillin, 100 μg/ml streptomycin (all from Gibco, Paisley, Scotland) at standard conditions of 5% CO_2_ and 37°C. Subculture of primary podocytes was performed after 6 days. Cellular identity was confirmed by detecting the expression of synaptopodin and WT1.

### Treatments with D-glucose and insulin *in vitro*

Freshly isolated Glomeruli or primary cultured podocytes were incubated for 24 h in medium HAM-F10 (glomeruli) and DMEM:F12 (podocytes) supplemented with 5 mM or 25 mM D-glucose in standard conditions of 5% CO_2_ and 37°C. To evaluate the effect of insulin on nucleoside transport activity, glomeruli or podocytes were exposed to 10 nM insulin for 30 min. To determine the effect of insulin on ENT1 and ENT2 protein expression levels, podocytes were exposed to 10 nM insulin for 48 h.

### Nucleoside transport activity

Freshly purified glomeruli or podocytes were incubated in 200 µl of choline solution (in mM: 5.4 KCl, 1.8 CaCl_2_, 1.2 MgSO_4_, 10 Hepes, 137 choline chloride, pH 7.4) supplemented with 1 µM NBTI or 2mM hypoxanthine for 30 minutes. Nucleoside transport activity was assayed in choline buffer supplemented with 1 µM NBTI or 2 mM hypoxanthine and 10 µM of adenosine containing 2,3[^3^H]-adenosine (2 μCi/nmol) for 30 seconds at 22 °C. Transport was stopped by washing with 1ml of cold buffer composed of 137 mM choline chloride and 10 mM Tris-Hepes (pH 7.4). Glomeruli were then centrifuged at 12000 × rpm for 5 minutes at 4 °C and washed again. The pellet or adherent podocytes were dissolved in 250µl of 0.5M HCOOH. Aliquots were sampled for protein determination and radioactivity counting^24, 25^. Particular uptake rates mediated by ENT1 or ENT2 were assigned to transport activities which were inhibited by 1 µM S-(4-nitrobenzyl)-6-thio-inosine (NBTI) or 2 mM hypoxanthine, respectively^39^. Total nucleoside uptakes in cells mediated by concentrative and equilibrative systems were also measured using transport buffer containing sodium chloride. Sodium-dependent uptake rates mediated by CNTs were obtained by subtracting adenosine uptakes in choline buffer to the total adenosine uptakes in buffer containing sodium chloride.

### Adenosine quantification

Glomeruli or cultured podocytes were incubated in Tyrode buffer (10mM HEPES, 12mM NaHCO_3_, 137mM NaCl, 2.7mM KCl, 5 mM Glucose and 1mM CaCl_2_) supplemented with 2.5µM EHNA and 1µM NBTI for 1 h. Adenosine content in the medium was quantified using derivatization with 2-chloroacetaldehyde and HPLC with fluorometric detection^24^. Values were normalized to total amount of cellular proteins.

### Western blots

Total proteins were extracted with lysis buffer (2% w/v SDS, 10% v/v glycerol, 63.5 mM Tris HCl, pH 6.8) containing Complete Proteinase Inhibitor and 1µg/ml pepstatin (Roche). Protein extracts (30µg) were fractionated by 10% w/v SDS-PAGE and transferred to PVDF membranes. Primary antibodies anti-ENT2 (ab181192, Abcam) and anti-ENT1 (11337-1-AP, Protein Tech) were used and detected with HRP-coupled secondary antibodies. A chemiluminescence procedure was used for detection of proteins (Thermo Scientific). The protein levels were expressed as ratio between target protein and β-actin detected in the same membrane.

### Animal Treatments

Adenosine deaminase from Bovine (Sigma) was PEGylated as previously described^43^. Following one month from diabetes induction in rats, adenosine deaminase was administered weekly by intraperitoneal injection of 5 U for a four weeks period. Control diabetic rats were administered with an equivalent volume of the vehicle PBS1X.

### Proteinuria

Urines of rat were recollected in metabolic cage for 24 h and protein contents were quantified by the pyrogallol red molybdate method (Proti U/LCR, Wiener Lab).

### Histological analysis

Rat kidney tissues were fixed in formalin, then paraffin embedded and 5 μm sections were mounted on silanized slides. Immunodetections were performed using primary anti-α-SMA (SC-130617, Santa Cruz Biotechnology) and anti-nephrin (AF3159, R&D System) in blocking solution overnight at 4°C. Immunosignals were developed using the LSAB + System − HRP (DakoCytomation). Images were analyzed by quantifying glomeruli in renal cortex using the software ImageJ with Color-Deconvolution plugin.

### Statistical analysis

Values are means ± SD, where *n* indicates the number of animals. Comparisons between two and more groups were performed by means of the unpaired Student t test and two way ANOVA, respectively. If the ANOVA demonstrated a significant interaction between variables, post hoc analyses were performed by the multiple-comparison Bonferroni correction test. *P* < 0.05 was considered statistically significant.

## Electronic supplementary material

Supplementary data
