# Author Correction: Deficient Insulin-mediated Upregulation of the Equilibrative Nucleoside Transporter 2 Contributes to Chronically Increased Adenosine in Diabetic Glomerulopathy

**DOI:** 10.1038/s41598-020-60880-z

**Published:** 2020-03-04

**Authors:** Sebastián Alarcón, Wallys Garrido, Génesis Vega, Claudio Cappelli, Raibel Suárez, Carlos Oyarzún, Claudia Quezada, Rody San Martín

**Affiliations:** Institute of Biochemistry and Microbiology, Science Faculty, Universidad Austral de Chile, Valdivia, Chile

Correction to: *Scientific Reports* 10.1038/s41598-017-09783-0, published online 25 August 2017

Génesis Vega was omitted from the author list in the original version of this Article. This has been corrected in the PDF and HTML versions of the Article, and in the accompanying Electronic Supplementary Material.

The updated Author List now reads:

Sebastián Alarcón, Wallys Garrido, Génesis Vega, Claudio Cappelli, Raibel Suárez, Carlos Oyarzún, Claudia Quezada, Rody San Martín

The Author Contributions section now reads:

Conceived and designed the experiments: SA, CO, CQ, RSM. Performed the experiments: SA, WG, GV, CC, RS, CO. Analyzed the data: SA, WG, GV, CC, RS, CO, CQ, RSM. Contributed reagents/materials/analysis tools: SA, WG, CC, RS, CO, CQ, RSM. Wrote the paper: SA, CQ, RSM.

