# Supplementary material for: Deficient Insulin-mediated Upregulation of the Equilibrative Nucleoside Transporter 2 Contributes to Chronically Increased Adenosine in Diabetic Glomerulopathy
Source: Sci Rep. 2017 Aug 25;7:9439. doi: 10.1038/s41598-017-09783-0 (PMC5572683; doi:10.1038/s41598-017-09783-0)
Supplement: Supplementary file 1 — Supplementary data [file 41598_2017_9783_MOESM1_ESM.pdf]

DEFICIENT INSULIN-MEDIATED UPREGULATION OF THE EQUILIBRATIVE NUCLEOSIDE TRANSPORTER 2 CONTRIBUTES TO CHRONICALLY INCREASED ADENOSINE IN DIABETIC GLOMERULOPATHY.

Sebastián Alarcón, Wallys Garrido, Génesis Vega, Claudio Cappelli, Raibel Suárez, Carlos Oyarzún, Claudia Quezada, Rody San Martín.

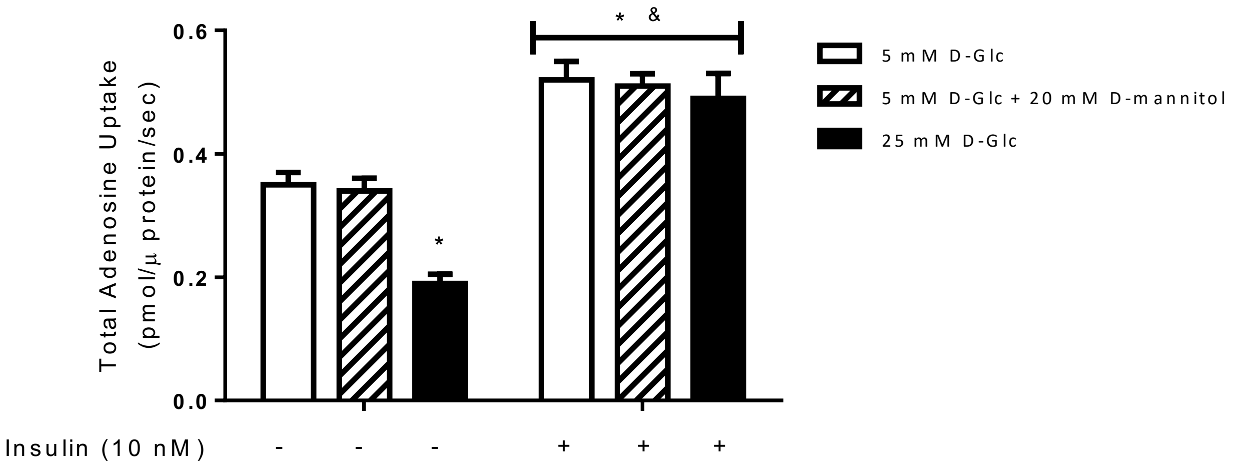

Supplementary Figure S1. D-glucose and insulin affect total adenosine uptake in rat glomeruli. Total adenosine uptake (10 µM adenosine, 60 seconds, 22ºC) mediated by sodium-independent transporter systems was measured in purified glomeruli from healthy male rats exposed to 5mM or 25 mM D-glucose (D-Glc) for 24 h and supplemented with insulin the last 30 minutes. Osmotic control using 5mM D-glucose supplemented with 20 mM D-mannitol was included. The graph represents the means ± S.D. *, $P < 0.05$ versus 5 mM D-Glc; $^{\&}$, $P < 0.05$ vs without insulin; n = 5.

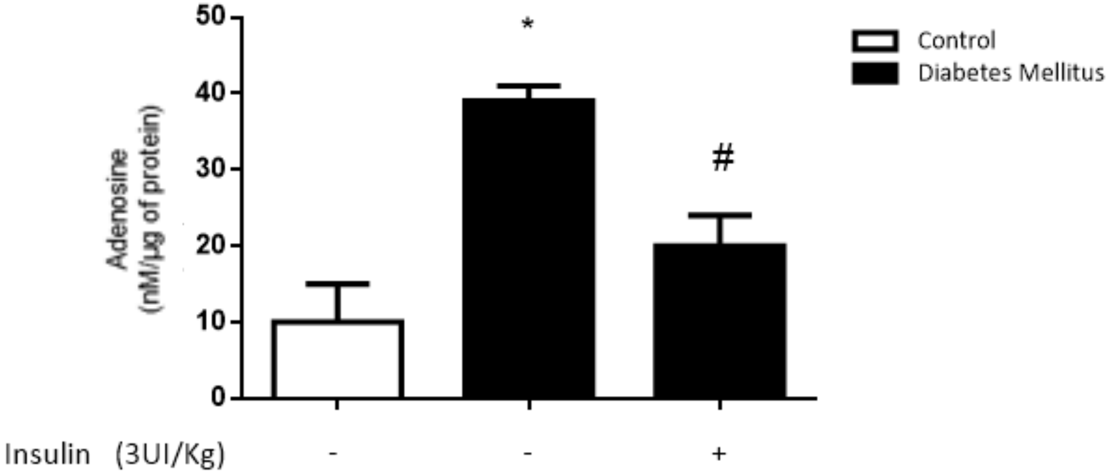

Supplementary Figure S2. Insulin replacement therapy reverses increased adenosine levels in diabetic rat glomeruli. STZ-induced diabetic rats were treated with 3UI/Kg insulin daily for 1 month. Glomeruli were isolated from rats and extracellular adenosine levels were quantified by using derivatization with chloroacetaldehyde and HPLC. Values are means ± S.D. from individuals determinations normalized to 1 µg of total glomerular proteins. *, $P < 0.05$ versus Control; $^{\#}$, $P < 0.05$ versus DM; n = 6.
